# Every School Healthy: Creating Local Impact Through National Efforts

**DOI:** 10.1111/josh.12963

**Published:** 2020-11-12

**Authors:** Elizabeth Pufall Jones, Daniel P. Hatfield, Nico Connolly

**Affiliations:** ^1^ Center for Promise at Boston University, 621 Common wealth Ave Boston United States 02215; ^2^ Childhood Obesity 180, Tufts University, 75 Kneeland Street Boston MA 02111; ^3^ America's Promise Alliance, 1110 Vermont Avenue, N.W, Suite 900 Washington DC 20005

**Keywords:** school wellness, school health, community engagement, National Youth Serving Organizations

## Abstract

**BACKGROUND:**

Whole school, whole community, whole child (WSCC) approaches to education address contexts beyond school that influence young people's academic and life outcomes. These recommended approaches demand mobilization of an array of actors, but such mobilization is challenging. Little research has explored strategies for convening national experts to support local communities.

**METHODS:**

This paper presents a case narrative of Every School Healthy (ESH), a grant‐funded effort to support organizations/school districts in 6 communities building WSCC initiatives by engaging national youth development organizations as expert advisors to ESH and communities. A thematic analysis of the narrative yielded 3 key learnings.

**RESULTS:**

Three themes emerged as key learnings regarding implementation of national‐local initiatives: (1) baseline assessment of local communities should be conducted to identify opportunities for maximizing strengths; (2) national organizations must be flexible with the expertise they bring to the initiative; (3) national organizations should prioritize community‐cultivated solutions and meet communities where they are.

**CONCLUSIONS:**

The 3 themes presented in this case narrative offer insights for effectively mobilizing national organizations to support healthy, equitable school environments at the local level.

Wellbeing is a process^1^ whereby individuals actively interpret their surroundings to understand how their context and its available resources might support them to reach their goals, stay satisfied with life, and meet their psychological, social, and physical needs. However, many individuals face obstacles that inhibit their ability to access resources they need to achieve wellbeing.^2^ To achieve an equitable balance of resources so everyone has the opportunity to be healthy, implementation of cross‐context approaches have been promoted.^3^, ^4^ Whole school, whole community, whole child (WSCC) approaches support wellbeing by addressing contexts beyond school and engaging all aspects of young people's lives in designing school policies, practices, opportunities, and resources.^4^, ^5^ Such approaches require coordinated activation of developmental opportunities across all contexts of young people's lives.

Young people are embedded within a multilayered ecology of relationships, built and natural environments, and norms, policies, and practices,^6^ collectively termed a *youth system*.^7^ Positive developmental outcomes arise through the alignment of young people's strengths and needs with the assets and supports present in their youth system, creating a *supportive youth system*.^7^ From this theoretical perspective, development occurs through co‐action between young people and the multiple contexts in which they are embedded, and positive development arises from contextual alignment with their strengths and needs.

Young people's learning is fostered through relationships that respond to, acknowledge, and integrate their experiences across their youth systems.^5^ Recognizing this whole child perspective, leaders such as the US Centers for Disease Control and Prevention (CDC)^4^ and the Aspen Institute National Commission on Social, Emotional, and Academic Development^3^ recommended that learning settings draw upon community partners to align resources that support all aspects of young people's development.

Schools are just one context of the youth system, and myriad other contexts impact young people's educational and health outcomes. As Zaff et al. indicate, to impact developmental outcomes demands more than building new or better programs; instead, “[a]n initiative is needed that starts with the needs and strengths of young people and creates an interconnected system of supports linked across age, context, and time.”^8^ A whole‐child approach, which promotes equitable access to resources and positive health outcomes for all, demands mobilization of diverse actors representing different components of the youth system. However, such mobilization is challenging, particularly when stakeholders hold different priorities and view the system through different lenses.

Growing research has explored promising approaches at the individual community level for convening diverse stakeholders, building consensus about problems and their causes, and mobilizing stakeholders toward developing and implementing shared solutions.^9^, ^10^ At the national level, there is a similar need to mobilize diverse stakeholders toward integrated approaches to supporting young people.^11^, ^12^ Given the reach of national organizations, collaborative efforts among such stakeholders can have broad‐scale impact. However, despite recent calls for national‐level collaborative efforts to advance comprehensive school health locally,^13^ much of the research in this area focuses on how national partners coordinate with one another, rather than how they coordinate with local partners^14^ seeking to meet the diverse needs of young people, families, and schools. Little research has explored strategies for convening diverse national‐level actors to support local communities and advance a shared vision of whole child wellbeing.

In this paper, we document one such effort, Every School Healthy (ESH), that attempted to coordinate national and local organizations to implement WSCC components within communities. ESH brought together national organizations with content expertise working collaboratively with community‐based organizations and coordinating activities among diverse stakeholders with different priorities and perspectives. It hypothesized that cultivating collaborative partnerships between such national organizations and local communities could accelerate the achievement of youth developmental outcomes and provide lessons for similar collaborative partnership efforts in the future.

In fall 2017, America's Promise Alliance (APA), a national nonprofit organization, conducted outreach to organizations and leaders to identify and convene core partner organizations representing an array of perspectives in whole child health and education. Together, these partners would collaboratively lead a multi‐year initiative supporting WSCC efforts being implemented by local school‐community partnerships, or acceleration sites, and advance understanding of what it takes to implement such efforts successfully.

Through this process, APA became more familiar with the landscape of issues, dynamics, and stakeholders at the intersections of education and health. These insights led to APA inviting 5 leading national organizations to serve as core partners: ChildObesity180, Communities In Schools, FoodCorps, National Association of Community Health Centers, and Turnaround for Children. These organizations served as strategic thought partners, responsible for co‐designing the initiative and informing decision‐making. Core partners also offered technical expertise to acceleration sites through structured learning sessions and ad hoc capacity‐building. Whereas these organizations have expertise in distinct but related domains of whole‐child health, prior to this initiative the degree of partnership between them varied, with several having little or no history of collaboration. APA served as the central facilitator. This role involved compiling and distilling the experiences, perspectives, and expertise of the core partners and other key stakeholders to develop and organize the partnership's collective goals for the initiative. Table 1 summarizes the participants in this work.

**Table 1:** Participants in the Every School Healthy Initiative

	Definition
Every School Healthy Initiative	Aims to create national movement toward healthy and safe schools that advance young people's education and promote their social, emotional, physical, and mental health Invested $1.8 million in 6 communities to accelerate progress by promoting capacity building and cross‐site learning, where practitioners from local sites as well as youth, researchers, and school leaders were learning from one another, both sharing and adopting best practices Employed national communications and broad dissemination to further contextualize and amplify learnings related to collaborative solutions to transform school environments into spaces where youth can thrive
America's Promise Alliance	Washington, D.C.‐based organization that convenes nonprofits, businesses, community and civic leaders, educators, citizens, and young people to catalyze action to improve the lives and futures of America's youth nationwide.
Core Partners	Five national organizations that America's Promise Alliance convened to co‐lead and support the Every School Healthy initiative: ChildObesity180—https://www.childobesity180.org/ Communities In Schools—https://www.communitiesinschools.org/ FoodCorps—https://foodcorps.org/ National Association of Community Health Centers—http://www.nachc.org/ Turnaround for Children—https://www.turnaroundusa.org/
Acceleration Sites	America's Promise Alliance invested a total of $1.8 million across 6 community partners in 5 states that are spearheading efforts to create healthy schools: Adelante Mujeres (Forest Grove, OR)—Youth development program that provides girls ranging from 3rd to 12th grade with weekly afterschool sessions that develop their leadership potential, build strong cultural identity, and foster healthy lifestyles and academic success. Alive and Well Communities (St. Louis, MO)—Initiative to promote trauma‐informed practices so schools can be environments that support the health and wellbeing of staff and students. Better Together Central Oregon (Crook County, OR)—Partnership with 7 key community organizations and the Crook County School District to train staff in trauma‐informed practices, increase capacity in schools for mental health support, and amplify youth and community voice. FIT2gether (Cherokee County, SC)—District‐led initiative to catalyze a culture of health through student‐led teams at each school in the district that identify barriers to health and well‐being and design solutions. Partnership for Child Health (Jacksonville, FL)—An effort to integrate trauma informed and child rights‐based approaches to advance whole child health in schools in Jacksonville's Urban Core. Staten Island Partnership for Community Wellness (Staten Island, NY)—Partnership with North Shore schools and community partners to empower young people, parents, and faculty to advocate for healthy schools.
Center for Promise	The applied research institute of America's Promise Alliance, dedicated to understanding what young people need to thrive and how to create the conditions of success for all young people. The Center for Promise designed and executed a learning agenda for the Every School Healthy initiative and also offered technical assistance to the sites with research related questions.
Youth Engagement Consultant	Expert researcher in youth engagement who was engaged to provide deep technical assistance to all 6 of the acceleration sites to improve their capacity to authentically engage youth in the design and implementation of their efforts.

In 2017, with support from the Robert Wood Johnson Foundation, APA and the core partners launched the Every School Healthy (ESH) initiative. The team operated from the belief that solutions to inequities in health and education demand investment in local knowledge and decision‐making, and local change agents should spearhead action toward equity. To this end, ESH invested $1.8 million to advance work of 6 acceleration sites working to create healthy schools. The initiative aimed to identify the conditions, practices, and community‐led strategies that foster healthy, equitable school environments, and amplify those learnings through broad dissemination activities.

In January 2018, ESH distributed a request for proposals (RFP) from communities engaged in cross‐sector collaborations to increase wellbeing for youth in schools. From over 138 applications from 20 states, 6 communities were chosen to become acceleration sites, receiving up to $150,000/year for 2 years. Beginning in summer 2018, core partners, a youth engagement specialist, APA and the Center for Promise (CFP)—the research institute for APA—began engaging acceleration sites through in‐person and virtual modalities to address their needs and capitalize on their strengths to support their efforts.

This paper aims to crystallize key learnings from ESH that may help inform other efforts to mobilize national partner organizations in supporting local partners with comprehensive school‐based approaches to child health and education.

## METHODS

To support and understand the work happening across the initiative, CFP engaged a learning agenda.^15^ From spring 2018 to summer 2019, CFP used various data‐collection methods to examine the initiative's processes and strategies that might contribute to positive youth outcomes. As part of the learning agenda, findings gleaned from data at key junctures were analyzed, interpreted, and reported back to national and local partners to improve engagement and outcomes.^15^ The learning agenda focused on understanding the processes involved in complex national‐local partnerships of this nature as a means to alleviate health disparities and inequities. Although the ultimate goal of this initiative is for all young people to thrive, realizing this goal in a 2‐year grant period was unrealistic; thus, we focused on examining initiative processes. Data collected and analyses completed to answer learning agenda research questions contribute to this case narrative. Specifically, this paper presents themes generated from examining ESH's strategies, successes, and challenges related to mobilizing national partners to support local efforts.

We conducted a 2‐part analysis. First, we utilized a narrative strategy to consider the contextual factors that might influence actors in the initiative, their experiences, and outcomes.^10^ We examined experiences within the initiative that potentially fostered collaboration among the national organizations and contributed to the performance of the initiative overall. Using data collected for the learning agenda, we created a chronological narrative of the initiative, highlighting actions and inputs, along with obstacles faced and how the initiative overcame them. We then distilled themes related to the strategies that meaningfully furthered the initiative. Table 2 provides a summary of themes and data examined related to each point. By examining the ESH story, we hope to reveal generalizable takeaways that national initiatives, funders, and other organizations can apply to support similar efforts and the ultimate outcome of thriving youth.

**Table 2:** Table Describing the Key Learning Themes and the Data Contributing to These Findings

Theme	Data Collected
(1) Baseline assessment of local communities should be conducted to identify opportunities for maximizing strengths	Participant observations during site visits regarding needs, assets, and relationships Examination of acceleration site grant application, documenting the areas for which sites requested support and the opportunities for TA from core partners and any needs and strengths they identified within the narrative Needs assessment memos Midpoint evaluation memo Field notes taken during the October 2018 meeting to review site visits and design technical assistance opportunities Field notes from December 2019 meeting with core partners to review the lessons learned from participation in the initiative
(2) National organizations must be flexible with the expertise they bring to the initiative	Participant observations during site visits regarding needs, assets, and relationships Field notes taken during the October 2018 meeting to review site visits and design technical assistance opportunities Midpoint evaluation memo Field notes taken during the July 2019 convening, with particular attention to how sites responded to and reached out for support from core partners as well as the opportunities that core partners offered for support Field notes from December 2019 meeting with core partners to review the lessons learned from participation in the initiative Mid‐grant narrative reports from acceleration sites Westat Topline Process and Early Outcome Evaluation Report
(3) National organizations should prioritize community‐cultivated solutions and meet communities where they are	Semi structured interviews with core partners and acceleration sites during our mid‐point evaluation Semi‐structured interview with the youth engagement consultant regarding stakeholder engagement Midpoint evaluation memo Youth engagement memo Field notes from December 2019 meeting with core partners to review the lessons learned from participation in the initiative Mid‐grant narrative reports from acceleration sites Westat Topline Process and Early Outcome Evaluation Report

## RESULTS

Three themes emerged about the attributes of ESH that were critical to success and that might be similarly important for other efforts to mobilize national‐level organizations to support local communities:

- Baseline assessment of local communities should be conducted to identify opportunities for maximizing strengths.
- National organizations must be flexible with the expertise they bring to the initiative.
- National organizations should prioritize community‐cultivated solutions and meet communities where they are.

More broadly, these themes highlight important steps in moving national level partners from thinking about their role in the initiative as the top‐down providers of expertise, to the bottom‐up supports that help accelerate local efforts. The following sections outline these themes and the evidence underlying them.

### Key Learning 1: Baseline Assessment of Local Communities Should Be Conducted to Identify Opportunities for Maximizing Strengths

Core partners and APA conducted site visits to develop a better understanding of the acceleration sites, document their assets and strengths, and collaboratively identify areas for support. They used insights from these visits, plus information from other sources, to inform the design and implementation of technical assistance (TA).

In summer 2018, APA organized site visits to each acceleration site, with teams including APA staff, core partner staff, CFP staff, and the youth engagement specialist. Site visit agendas were codesigned by accelerations sites and APA to include opportunities to meet community and school partners, see the surrounding community, and observe programming. A field note template was created to document relationships, assets, and needs, both those identified by sites and those observed, within the acceleration site communities. Documented assets and needs from site visits were then compiled with the needs and assets identified by acceleration sites in their applications. The needs and assets were then organized by acceleration site and theme and shared with the acceleration sites, core partners, and APA staff as a validity check.

The core partners, APA staff, and CFP reconvened in fall 2018 to review the needs and assets analysis and other data collected during site visits and to discuss the content and delivery of TA to acceleration sites (see Table 3 for a summary of the needs and assets identified through this process). The meeting began with reflections from the visits. A transcript review from this portion of the discussion reveals that core partners reflected primarily on how their programs might fit with acceleration sites' needs that they perceived, and not necessarily the needs that the sites themselves identified. For example, one core partner indicated that a site was very “doable” for their intervention model, and that the site would be “a good entry point” for them into the site's home state. Another core partner indicated the need to “keep our bias in check” and not impose core partner frames onto the sites and instead help communities do what they wanted to do.

**Table 3:** Summary of Needs and Assets Themes and the Percentage of Acceleration Sites Identifying Each Theme within Their Community

Needs	%	Assets	%
Data Collection and Analysis/Program Evaluation	83	Partnerships	100
Youth Engagement	100	Youth Involvement	67
Community Engagement	67	Parent Involvement	33
School Engagement	67	Community Resources	33
Family Engagement	67	School Engagement	67
Operations	87	Philosophy and Vision Impact	100
Communications	33	Organizational Resources	83
Bias and Equity	83		
Alliance	50		
Nutrition	17		
Trauma	17		
Health Care	17		
Sustainability	17		

CFP and APA then recalibrated the discussion, framing the presentation of needs and assets by saying:

“…*we are presenting the data in this way because we want to keep the work site centric—it is not about what core partners can do for them but what the site needs and desires*.”

This statement prompted the core partners to think beyond their usual suite of materials to more broadly consider how they could meet acceleration site needs as opposed to their own. When CFP asked how the presentation of data influenced their thinking about TA, core partners indicated that seeing the needs and assets aligned thematically pushed them to think about finding ways to provide support across sites initially, and then base subsequent individual site TA on sites' responses. As one core partner said, they should “offer a suite of opportunities to build relationships with sites, then once the relationship is established go more in‐depth.”

Thus, whereas many core partners expected TA to begin immediately after this meeting, a tempered approach to building the support structure was important and dictated by the analyses of needs and assets. Although incorporating some formulation of asset mapping/needs assessment might be typical for complex initiatives of this nature, centering assets and needs of sites, as opposed to national organization agendas, to shape and lead the discussion is essential. Furthermore, creating shared understanding across all levels of the initiative about needs and assets can help ensure success.^16^

### Key Learning 2: National Organizations Must Be Flexible with the Expertise they Bring to the Initiative

Concluding the fall 2018 meeting, APA staff and core partners realized that TA content that core partners initially imagined delivering to sites was largely not needed. As one partner said during the meeting, diverging from “…the core competencies that [they brought] for the purposes of this project [feels] like coloring outside the lines.”

The core partners have experience in specific school‐based health and wellness activities, like creating physical activity programs or building partnerships between community health centers and schools. The site assets and needs analysis showed that specific content expertise often was not necessary because sites already possessed related expertise and/or capacity, or the core partner content expertise was not related to new activities sites proposed. Thus, the initiative faced a need to pivot from the previously conceived notion of tapping into existing core partner content expertise to a more nuanced interpretation of TA that would be useful to acceleration sites.

After reviewing the assets and needs analyses, core partners and APA staff reflected on their own work and potential intersections with the acceleration sites' efforts:

“*There was a very clear shift after the site visits and the meeting on October 1^st^, where it was clear that the expertise that we brought to the table was not the expertise needed by most of the sites*.”

“*We've pivoted given the mismatch in expertise to look more at the sites and how we can support them organizationally as opposed to with content*.”

Indeed, the core partners understood that a whole child approach requires that we look at our own strategies, processes, and practices in new ways. As one core partner stated:

“*[they] really tr[ied] to figure out and drill down, like what did they [the sites] need to accelerate? What do they need to do to take the next step in the direction they want?*”

Many of the core partners were flexible and pivoted when challenges arose due to the varied expertise they possess. Often this involved shifting to build capacities that complemented the local efforts already underway, as opposed to leveraging specific content expertise or an already established model.

For example, FoodCorps joined the partnership with extensive expertise in creating nutrition curricula in schools but subsequently realized acceleration sites needed support in other areas. FoodCorps also has significant experience in implementing and evaluating their programming, which matched with the need for research and evaluation support identified by all acceleration sites. Thus, FoodCorps pivoted and offered a session on evaluation‐informed program design to sites. Similarly, ChildObesity180 joined ESH with expertise in school‐based programming around healthy eating, physical activity, and obesity prevention but found that sites either were not focused on these issues or already had relevant programming in place. However, several sites expressed challenges with mobilizing community stakeholders around health‐related challenges and devising shared solutions. ChildObesity180 has experience using Group Model Building (GMB) in community intervention work and has found that many challenges related to building cross‐sector partnerships can be addressed using this approach. Therefore, they engaged multiple acceleration sites in virtual and in‐person GMB workshops where they introduced the approach and provided hands‐on opportunities to apply core concepts.

Thus, when organizing initiatives and recruiting organizations to participate at both local and national levels, a requirement should be flexibility and responsiveness in terms of engagement. Rather than offering prescriptive support, both local and national partners need to think about all the assets they possess and how these might be modified to best support work of a given community. Flexibility like that exhibited by these core partners enabled them to “color outside the lines” and provide sites relevant, timely support.

### Key Learning 3: National Organizations Should Prioritize Community‐Cultivated Solutions and Meet Communities Where They Are

Throughout this initiative, national partners sought to authentically engage stakeholders as agents of change. Authentic engagement requires sharing power with stakeholders, allowing their voices to shape the direction of the work that is done *with* them rather than *for* them.^17^, ^18^, ^19^ For example, given ESH's focus on increasing youth voice, the Staten Island Partnership for Community Wellness consulted the youth engagement specialist to help formulate a youth participatory action research study where young people designed the protocol, collected data, and conducted analyses to assess their community's challenges. In addition, whereas the initiative prioritized the authentic engagement of all stakeholders, there was an exceptionally enthusiastic embrace of the youth engagement work.

The phrase “meeting the sites where they are” is a way of thinking about how to build interventions *with* rather than *for* a site. In this way ESH tailored support to each acceleration site's unique circumstances, building upon the initial analyses of assets and needs, pivoting as the initiative progressed and APA and core partners learned more about sites and/or events that occurred in the communities.

Tailoring support in this way meant that not every core partner engaged significantly with an individual site. Although some might view this as a shortcoming, many of the sites appreciated the flexibility in TA requirements as it alleviated redundancies and overinvestment of scarce resources. As one site stated, they appreciated that the grant:

“*didn't require a ton of meaningless connection just to fulfill grant requirements…[we were not] forced into engagement with core partners…and [were instead] able to pick and choose*…”

Rather than imposing artificial interventions on sites in a top‐down, prescriptive fashion, APA and core partners were responsive to acceleration sites, supporting them from the bottom‐up, working with sites to decide how best to allocate resources so their efforts might accelerate. As another site stated, designing the intervention in this way.

“…*allowed us to continue doing what we were already doing without compromising on our values*.”

Engaging authentically with stakeholders, understanding their unique circumstances, and prioritizing the needs and strengths present in communities also help to advance health equity.^2^ To understand health inequities that exist, we might examine data about certain conditions in a community, but how those conditions are processed, understood, and embodied is extremely personal.^2^ Furthermore, by sharing power with those who are proximate to inequities, national organizations can help promote a culture of health^2^ for all. In this way, local change agents can act as trusted messengers to lead conversation and action on promoting health equity across systems. The examples within this narrative offer a glimpse into ways in which stakeholders (communities, youth) told their own story, designed their own solutions, and were involved in guiding the initiative.

## DISCUSSION

A large body of research evidence and policy recommendations have highlighted the importance of collaborative partnerships among diverse stakeholders to advance comprehensive school health initiatives. Whereas research around such collaborations has focused largely on local‐level efforts,^20^ national‐level collaborations have also been shown to be important. In 1997, for example, the NIH report “Schools & Health: Our Nation's Investment” highlighted the promise of coordinated action at the national level.^14^ That report pointed to the success of national initiatives like the National Coordinating Committee on School Health and Safety, which was formed to convene national non‐governmental organizations to support comprehensive school health. Other more recent papers have called for continued efforts to promote and optimize national‐level collaborative efforts to advance comprehensive school health locally.^13^

Despite recognition of the promise in mobilizing national collaborations to support local efforts, relatively few studies have described how such efforts can be effectively executed or outlined lessons learned from what work has been done, leading some authors to call for new research describing national partnerships that might serve as models.^12^ This is one of few papers to describe an initiative to bring together leading national organizations in school health to support local communities, including learnings from that experience that may be instructive for other similar initiatives. To our knowledge, it is the only such paper to describe an initiative through which national organizations engaged directly with local partners, as opposed to working indirectly through national‐level advocacy, provision of guidelines, or other broad‐based approaches.

Our findings about factors that activated the success of this work are similar to those observed in other studies related to collaborative partnerships. For example, prior studies focusing on local‐level collaborative partnerships to promote whole‐child health have highlighted the importance of conducting baseline assessments to understand organizational needs and opportunities^21^ and of engaging a variety of stakeholders in developing and implementing of health‐related school initiatives.^22^ We also find that conducting baseline assessments and “meeting communities where they are” are critical for national‐level partnerships with similar goals for advancing comprehensive school health locally. Likewise, our observation about the importance of flexibility in this national level collaborative work is similar to Potts‐Datema et al.'s conclusion about the need to “[b]e nimble and able to change course if the need arises” in other national‐level partnerships.^23^ Whereas that observation related chiefly to flexibility in response to national‐level developments (eg, nimble responses to changing Congressional agendas), we find that flexibility in response to shifting local needs is similarly paramount for national organizations engaging directly with local partners.

We recognize the importance of engaging state‐level policymakers to support policy shifts that catalyze adoption of comprehensive school health efforts, and we believe that state‐level policy should be responsive to and guided by the experience of local stakeholders. However, the work of this initiative centered mostly on national partners directly engaging local communities through a unique partnership structure rather than engaging state‐level policymakers. With that said, acceleration sites advanced comprehensive school health by demanding and helping to guide policy change at state and local levels. For example, one site prepared guidance for schools wanting to become trauma‐informed that was adopted by their state board of education. Building upon that work, the acceleration site and its partners decided to pursue a state statute requiring school districts to have a policy on being trauma‐informed.

A lynchpin to the success of this work was the central facilitating role played by APA, including activities like organizing baseline assessments and guiding adaptations based on shifting local needs. Other studies have likewise noted the critical importance of centralized leadership, including funding, dedicated staff, and organizational support, to enable success of national‐level collaborative work to support comprehensive approaches to school health.^23^

This case narrative captures the systematic study of one, unique initiative attempting to coordinate national partners to accelerate local community work. To improve understanding of how themes presented in this case manifest more generally, it is important to study them across a variety of different initiatives doing similar work. Furthermore, these themes broadly describe the strategies and processes important to the success of the initiative, rather than a formula of particular strategies and processes to ensure success. We offer these themes as guideposts in particular for national organizations interested in pushing beyond their commonly held expectations so that they might authentically engage with local partners. Only in doing so will they be able to encourage positive outcomes in local communities.

## IMPLICATIONS FOR SCHOOL HEALTH

Collaborative partnerships across community and national levels are an important component to successfully implementing WSCC approaches in schools. Although they do not necessarily move individual student outcomes, they do provide the conditions at the partnership level of youth‐serving systems, like education, that support the impact of whole child efforts. Amidst an environment of limited resources and shrinking budgets, schools and community organizations that support schools often seek national‐level partnership to strengthen capacity to support the whole child within schools. The resources, access to information, and expertise that national partners offer can strengthen school‐based efforts while providing funds and opportunities for schools and community partners to innovate. The experience of this initiative indicates that schools and community organizations are able to cultivate community‐led solutions when they are not working with overbearing national partners primarily interested in prescriptively implementing a specific program, intervention, or model. National initiatives seeking to work with community partners should, beyond other considerations, strive to meet communities where they are by aligning initiative structures and practices toward a vision of flexible support between all partners.

Schools and community organizations should therefore seek out and communicate clearly with national partners that embrace flexibility and provide room for pivoting, especially if unexpected changes occur within a school or community. These flexible and adaptive partnerships are fostered when local and national partners have taken time to build relationships and trust through activities like site visits, in‐depth needs and assets assessment, and regular, transparent communication. Familiarity with community realities and context can help national partners know what kinds of resources are helpful to local partners and, equally important, which ones might not be at any given time. Similarly, engagements between national partners and community partners like those in this initiative provide opportunities for national organizations that target school health to strengthen their own work by experiencing and interacting with work happening in communities from a different perspective than that to which they might be accustomed.

Supportive and flexible national‐local partnerships can allow community partners to adapt their work in response to unexpected and disruptive changes at the community level. Unexpected changes experienced locally in this initiative included externalities like district leadership transitions, a political climate that caused anxiety and fear in Latinx communities, and impacts of community violence on the lives of local young people involved with the work. By using an approach where sites had access to support from core partners if they needed it, but were not required to do work with core partners that might not have been closely aligned with emergent priorities, sites were supported in their efforts to drive impact and navigate through the types of unexpected shifts that are common in the US education system.

### Human Subjects Approval Statement

This research was considered exempt by the Boston University Institutional Review Board as it was not considered human subjects research and did not present an increased risk to any of the participants.

### Conflict of Interest

All authors of this article declare they have no conflicts of interest.
